# Endoscopic diagnosis of primary anorectal melanoma

**DOI:** 10.18632/oncotarget.15495

**Published:** 2017-02-18

**Authors:** Sheng Wang, Siyu Sun, Xiang Liu, Nan Ge, Guoxin Wang, Jintao Guo, Wen Liu, Shupeng Wang

**Affiliations:** ^1^ Endoscopic Center, Shengjing Hospital of China Medical University, Shenyang, China

**Keywords:** anorectal melanoma, colonoscopy, endoscopic ultrasound

## Abstract

**Objective:**

The present study retrospectively analyzed case data from 12 patients diagnosed with anorectal melanoma, with the purpose of identifying key diagnostic features at endoscopy.

**Materials and Methods:**

Images from colonoscopy were reviewed for all patients in order to establish the endoscopic features of primary anorectal melanoma. For the patients whose colonoscopic examinations included endoscopic ultrasound, images were examined to characterize lesions and the depth of infiltration, the results of which were compared with pathological findings after operative resection.

**Results:**

At colonoscopy, superficial melanin pigmentation was identified in 10 patients with anorectal melanoma, with morphology including spots, patches, or sheets of pigmentation. In patients who underwent endoscopic ultrasound, lesions appeared as masses on the mucosal side with inhomogeneous or low-level internal echoes or ulcer-type lesions invading the muscularis propria. Lesions diagnosed as anorectal melanoma also demonstrated irregular margins and varying degrees of submucosal infiltration. Infiltration depth of melanoma *via* endoscopic ultrasound (EUS) was concordant with surgical pathology results in 100% of patients.

**Conclusion:**

Colonoscopy combined with biopsy and subsequent pathological examination can accurately diagnose primary anorectal melanoma. Moreover, EUS is a reliable tool for assessing the depth of infiltration of this disease.

## INTRODUCTION

Melanoma, which predominantly originates in the skin, has experienced a dramatic increase in public awareness, mirroring the rising incidence of its cutaneous form [1]. On the other hand, gastrointestinal (GI) tract melanoma is frequently overlooked, due in part to its lower frequency, occult location, lack of clearly-defined risk factors, and nonspecific, often confounding symptoms [2, 3]. In particular, primary anorectal melanoma, while accounting for only about 1% of melanoma diagnoses and less than 0.5% of anorectal cancers, nonetheless remains the 3^rd^ most common pathogenic site in melanoma, and is generally associated with poor prognosis [8, 9].

Currently, the diagnosis of primary anorectal melanoma is most frequently established *via* endoscopy with biopsy and immunohistochemical staining. Early diagnosis may facilitate definitive resection, which has been demonstrated to prolong disease-free survival in several studies [4–7]. Nevertheless, the common failure to reach this diagnosis in the early stages of disease contributes to the high incidence of locally advanced or widely metastatic disease at the time of diagnosis, and may often be partially attributable to limited awareness and/or infrequent consideration on the part of clinicans.

Extensive prior investigation has established the use of colonoscopy and endoscopic ultrasound (EUS) in the diagnosis and staging of anorectal carcinoma. However, there has been limited dedicated evaluation of these modalities in the workup of anorectal melanoma. Establishing reliable imaging features of the disease could simplify this typically challenging diagnosis, particularly for less-experienced clinicians, thereby offering the potential to increase identification of anorectal melanoma in earlier clinical stages.

The present study retrospectively analyzed case data from 12 patients with anorectal melanoma treated in our hospital between 2008 and 2015, including detailed characterization of images from colonoscopy and EUS. All the 12 patients were treated for the first time in our hospital. The imaging and pathological findings in our patients were examined in combination with review of the prevailing literature regarding the use of these modalities in the setting of melanoma and other anorectal cancers. Our analyses were performed with the purpose of identifying consistent endoscopic features to aid in the diagnosis of primary anorectal melanoma, as well as to observe and evaluate the depth of invasion, provide a diagnostic tool for the staging of rectal melanoma, which is benifit for predicting surgical resectability and prognosis.

## MATERIALS AND METHODS

### Patient population

We identified patients diagnosed with primary anorectal melanoma at our institution between August 2008 and July 2015, confirmed by colonoscopy, biopsy, and surgical pathology. The patients' charts were reviewed for gender, age at presentation, symptoms at presentation, and the duration of symptoms prior to presentation. The mean age at diagnosis and the mean duration of symptoms prior to presentation were calculated and reported as mean (range).

### Colonoscopy

All colonoscopic examinations were carried out with Pentax colonoscopes (EC-3890FI and EC 3870FZK) utilizing the EPKi processor (Pentax, Tokyo, Japan). Images from colonoscopy were examined and characterized in all patients to establish the endoscopic features of anorectal melanoma.

Lesions detected at colonoscopy were characterized for morphology, defined as lump-type, ulcer-type, or combined lump-and-ulcer type. The appearances of lesions were also recorded, including their apparent origins, margins, coloration, and surface characteristics.

Each lesion identified at colonoscopy was evaluated for the presence of superficial melanin pigmentation. The shape of melanin pigmentation, when present, was characterized as a spots, patches, or sheets.

### Endoscopic ultrasound

During colonoscopic examinations with forceps biopsy, EUS was utilized in several patients to improve characterization of lesion size, level, and depth of invasion within and beyond the bowel wall. All EUS examinations were carried out using a Pentax EG-3870UTK echoendoscope (Pentax, Tokyo, Japan) with a linear-array transducer, in combination with a Hitachi EUB-6500 ultrasound machine.

Endoscopic ultrasound, through observing the different echo of lesion and the surrounding normal tissue, can measure the diameter and thickness of the lesion, observe the various levels of the intestinal wall, determine the depth of invasion of the lesion. The location of lesions in relationship to the dentate line was carefully examined.

### Histopathologic evaluation and diagnosis

Pathological analysis of biopsied tissue included conventional histopathological examination, supplemented with HMB-45, S-100, and Vimentin immunohistochemical stains.

We recorded the final diagnosis rendered for each patient, as well as the modalities used to establish the diagnoses. For patients who underwent both EUS and eventual tumor resection, findings on surgical pathology were examined to assess for concordance with T-staging performed with EUS.

## RESULTS

### Patient population

Between August 2008 and July 2015, a total of 12 patients who underwent both endoscopy and biopsy received a diagnosis of primary anorectal melanoma confirmed by pathology. Of these patients, 4 were males, while the remaining 8 were females, with a mean age of 54.5 years (range = 47-65 years) at disease onset. In order of decreasing frequency, the most common symptoms at presentation in our patient population were as follows: hematochezia (9 patients), mass prolapsing through the anal canal or rectum (2 patients), and anal discomfort (1 patient). Patients presented on average 3.2 months after onset of symptoms (range = 0.2-10 months).

### Colonoscopy

**Figure 1:**
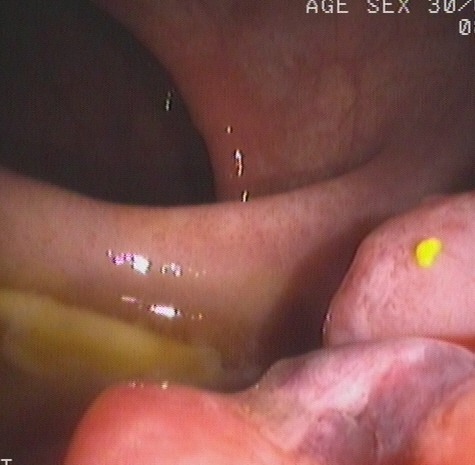
Image from colonoscopy demonstrating a lump-type lesion with a wide base and rough surface in the rectum near the dentate line An ulceration and large, black patches were present on the surface of the lesion.

**Figure 2:**
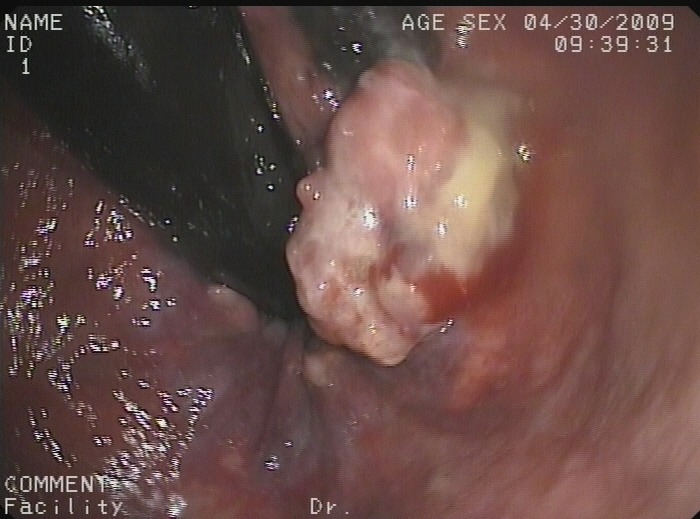
Image from colonoscopy revealing a lump-type lesion with a rough surface and black patches projecting from the mucous membrane near the dentate line

All patients underwent colonoscopy (Figures 1 and 2), revealing lump-type lesions in 8 patients, ulcer-type lesions in 1 patient, and combined lump-and-ulcer-type lesions in 3 patients. Superficial melanin pigmentation was identified within lesions discovered on colonoscopy in 10 patients, with pigmentation taking the shape of spots, patches, or sheets. There was no observable melanin pigmentation on the surface of lesions in the remaining 2 patients who were ultimately diagnosed with anorectal melanoma at pathology. All lesions invaded the dentate line, with propensity to the rectum in 7 patients, the dentate line as the midpoint in 3 patients, and propensity to the anal canal in 2 patients.

### Endoscopic ultrasound

**Figure 3:**
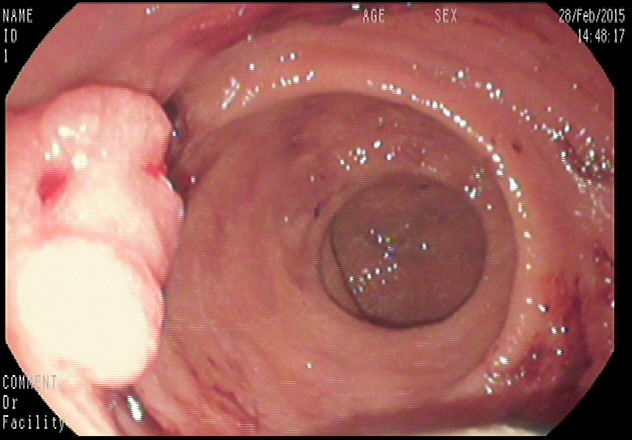
Image from colonoscopy demonstrating a lump-type lesion in the rectum with a rough surface, wide base, and local ulceration

**Figure 4:**
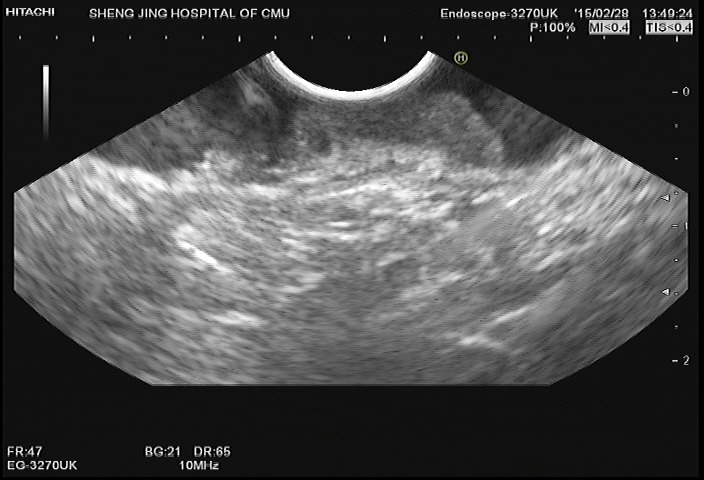
Image from EUS obtained for evaluation of a lesion identified on colonoscopy The EUS probe was placed to the lesion. The image demonstrates abnormally decreased echogenicity and thickening of the mucosa and submucosa. The muscularis propria is completely visualized.

**Figure 5:**
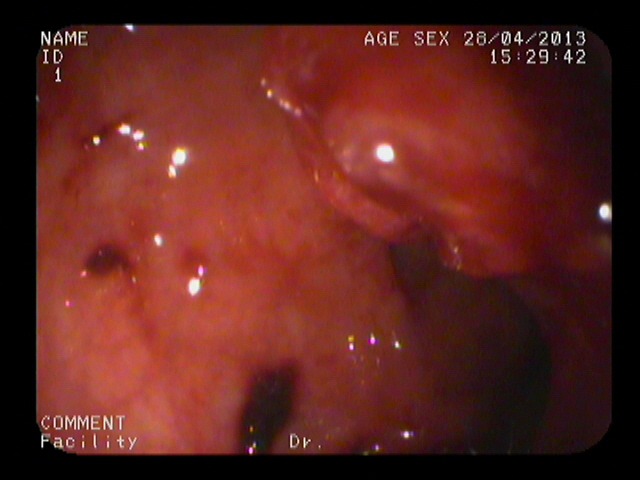
Image from colonoscopy demonstrating a lump-type lesion near the dentate line with a wide base and rough surface Ulceration and black patches were noted on the surface of the lesion.

**Figure 6:**
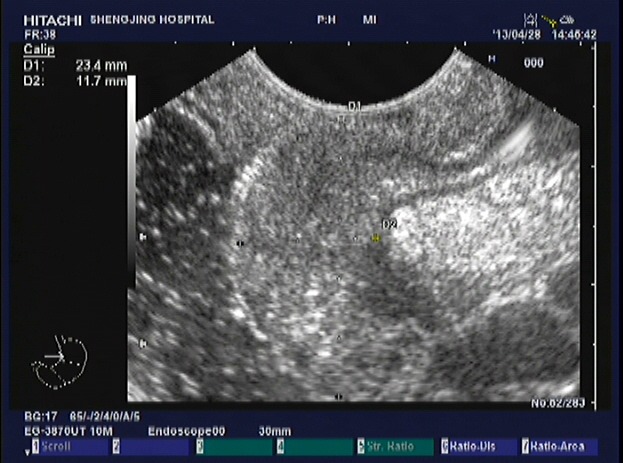
Image from EUS performed in the same patient revealing abnormally thickened and hypoechoic mucosa and submucosa The third layer is incomplete. There is also abnormal thickening of the subserosa.

A total of 5 patients underwent EUS in order to characterize the depth of invasion in lesions identified on colonoscopy (Figures 3-6). Mean lesion size measured 3.4 cm (range = 2.5-4.0 cm). All of the lesions imaged with EUS appeared as masses originating in the mucosa with inhomogeneous or low-level internal echoes. Lesions also consistently exhibited irregular margins, as well as varying degrees of submucosal infiltration. Ulcer-type lesions tended to invade the muscularis propria.

EUS revealed invasion of the submucosa in 2 patients. Lesions evaluated in the remaining 3 patients exhibited invasion of the muscularis propria. None of the 5 patients imaged with EUS demonstrated locally enlarged lymph nodes in the imaged field of view.

### Histopathologic evaluation and diagnosis

The correct pathological diagnosis of anorectal melanoma was identified *via* colonoscopy in 10 patients. The remaining 2 patients, who were initially diagnosed with rectal carcinoma *via* colonoscopy, were definitively diagnosed with primary anorectal melanoma. One case was diagnosed by pathological examination after colonoscopic biopsy, the other case was diagnosed by pathological examination after surgery, although the initial result of colonoscopic biopsy is colonic carcinoma.

Finally, immunohistochemical examination with HMB-45, S-100, and Vimentin staining was positive for melanoma in all patients with pathological diagnosis of anorectal melanoma. Furthermore, in all 5 patients who underwent both EUS and surgical resection, the depth of infiltration established by EUS was concordant with findings at surgical pathology.

## DISCUSSION

Compared to cutaneous melanoma, GI tract melanoma is exceedingly rare, most commonly originating in the rectum and anal canal, followed by the stomach, small intestine, and colon [10–12]. Yet, anorectal melanoma alone accounts for over 50% of primary GI tract melanomas [2, 4, 13]. Despite a relatively wide age range, anorectal melanoma is most frequently diagnosed in patients older than 50 years of age, and more commonly in females than in males [14, 15]. Concordantly, our study observed a mean age of 54.5 years at the time of diagnosis, with females representing 8 of the 12 patients in our study with primary anorectal melanoma.

At present, the diagnosis of primary anorectal melanoma is most commonly reached using a combination of colonoscopy and pathological examination [12, 16]. Colonoscopy alone proved effective in our study in the identification and characterization of anorectal lesions, establishing the correct diagnosis of primary anorectal melanoma in 10 of the 12 patients we investigated. The failure to identify characteristic melanin pigmentation in lesions from 2 patients in our study with anorectal melanoma may be due to melanoma can be expressed as surface dark spots, brown spots and no stain, so case without is easily diagnosed as colonic carcinoma by colonoscopy. However, the combination with pathological biopsy could increase the positive rate to 11/12 in our diagnosis. The conclusion of image features in the detected patient cases revealed superficial melanin pigmentation in lesions and lesions invading the dentate line, which should be the main basis for the suspicion of anorectal melanoma. When we can not diagnose melanoma with surface dark spots, we can improve the diagnostic rate with pathological and immunohistochemical stain. This lesion's endoscopic appearance was not very typical and conventional pathology diagnosed the lesion as poorly differentiated adenocarcinoma. However, the postoperative pathology made the final diagnosis as melanoma. Through this case, we suggest we shoud do multipoint biopsies to avoid misdiagnosis. We will talk about it in discussion section.

Prior investigations of the performance of EUS have demonstrated its ability to reliably differentiate the five layers of the bowel wall [17–19], as well as its attendant efficacy in the accurate T-staging of tumors of the GI tract [20–22]. A previous case report detailing the use of EUS and MRI in the diagnostic evaluation of a patient with anorectal melanoma proposed the utilization of these modalities as staging tools in the preoperative setting, similar to their applications in other rectal tumors [23]. In our present study, EUS was utilized to examine anorectal melanoma and supplement colonoscopic imaging and biopsy. We observed outstanding performance of EUS in the the depth of infiltration of anorectal melanoma, with 100% accuracy of our findings as measured against postoperative surgical pathology. However, despite these encouraging results, our relatively small sample size limits the statistical power of our investigation. Consequently, dedicated evaluation of colonoscopy and EUS in the diagnosis and T-staging of primary anorectal melanoma in larger patient populations is warrented to elucidate the significance and implications of our analysis. For the disease involving the dentate line, the application of radial scan endoscope will compress the lesion, affecting the observation. Convex array probe in linear array endoscope can be used to push the lesion slightly into the rectum, which can reduce the compression and facilitate observation with water.

In its current state, the limited scanning range of EUS hinders its application to evaluate the lymph node metastasis of anorectal melanoma. Nonetheless, its precise structural characterization of the bowel wall may eventually establish EUS as an indispensable tool in the of this disease [24–26]. Moreover, the option of immediate EUS-guided biopsy of suspicious perilesional lymph nodes identified during an examination offers an advantage over MRI in assessing the locoregional extent of disease [27–29]. Currently, there is no standard staging of colorectal melanoma such as the colorectal cancer, but the endoscopic ultrasound can accurately assess the depth of the infiltration of lesions, which will help the future staging.

Pathological evaluation, particularly in combination with immunohistochemical techniques, plays an integral role in the definitive diagnosis of primary anorectal melanoma [12]. The general lack of awareness of the disease on the part of clinicians and subsequently limited understanding of the ideal diagnostic approach likely contribute to the relatively lower sensitivity of colonoscopy in the diagnosis of primary anorectal melanoma. Indeed, an insufficient amount of tissue acquired *via* biopsy and incomplete tissue characterization are frequently implicated in the failure to diagnose this disease. Establishing reliable imaging features of anorectal melanoma could potentially facilitate improved diagnostic proficiency of clinicians. Identification of these imaging findings might thereby prompt the acquisition of larger-volume tissue samples through multi-dot or multi-block biopsies, which could improve both the sensitivity and accuracy of colonoscopy in the diagnosis of this condition.

Currently, surgical management, when feasible, is the preferred definitive therapeutic option for primary anorectal melanoma. On the other hand, chemotherapy, radiotherapy, and immunotherapy, when used, typically assume complementary roles, usually in neoadjuvant/adjuvant or palliative applications [29–32]. Regardless of therapy, the prognosis of anorectal melanoma overall remains poor, with a 5-year survival rate of less than 20% [33, 34]. This dismal prognosis, which predictably correlates with the stage of disease, is exacerbated by the occult location of lesions, absent or nonspecific symptoms, and common extensive local invasion, all of which contribute to the frequent intial presentation of patients in advanced stages of disease, often with distant metastases [12]. Furthermore, locoregional resection of anorectal melanoma, generally reserved for earlier stages of disease, has displayed a clear survival benefit over non-operative therapy, further emphasizing the importance of early detection of disease.

In addition to routine physical examination with digital rectal exam, thorough endoscopic evaluation can be a essential component in the early diagnosis of anorectal melanoma, particularly in combination with EUS to facilitate rapid, reliable tumor staging. However, the optimal endoscopic approach to diagnose anorectal melanoma varies slightly from that employed for more common anorectal cancers, including, for example, acquiring larger-volume tissue samples and applying specific immunohistochemical stains. Tailoring the examination to the diagnosis of anorectal melanoma requires first identifying imaging features that heighten suspicion for this rare clinical entity. Therefore, delineating consistent endoscopic features of melanoma represents a vital step toward improving the early detection and subsequent treatment of this disease.

In summary, for the assessment of anorectal lesions identified on colonoscopy, we propose the presence of superficial melanin pigmentation and invasion of the dentate line as potential diagnostic indicators of primary anorectal melanoma. When identified, these endoscopic findings should prompt the acquisition of sufficient volumes of samples tissue to permit both standard pathological and directed immunohistochemical investigation. In selected patients, EUS can accompany the colonoscopic examination to accurately characterize the tumor and the extent of local invasion.

## CONCLUSIONS

Colonoscopy combined with biopsy and subsequent pathological examination can accurately diagnose primary anorectal melanoma. Moreover, EUS is a reliable tool for of this disease.
